# Feasibility of noninvasive near-infrared spectroscopy monitoring in predicting the prognosis of spontaneous intracerebral hemorrhage

**DOI:** 10.3389/fneur.2024.1406157

**Published:** 2024-07-24

**Authors:** Zhen Sun, Jing Liu, Kunpeng Wang, Jiandang Zhang, Sujie Liu, Fei Xue

**Affiliations:** ^1^The Neurosurgery, Nanyang Central Hospital, Nanyang, Henan, China; ^2^Zhujiang Hospital, Southern Medical University, Guangzhou, Guangdong, China; ^3^Shaanxi Provincial People's Hospital, Xi'an, Shaanxi, China

**Keywords:** spontaneous intracerebral hemorrhage, intracranial pressure, cerebral oxygen metabolic indexes, combined prognosis prediction, near-infrared spectroscopy

## Abstract

**Objective:**

This study aimed to assess the impact of multimodal monitoring on predicting the prognosis of patients with spontaneous intracerebral hemorrhage (SICH) and to examine the feasibility of using noninvasive near-infrared spectroscopy (NIRS) for monitoring clinical prognosis.

**Methods:**

Clinical data of 38 patients with SICH who underwent surgery in the Department of Neurosurgery of Shaanxi Provincial People’s Hospital from May 2022 to December 2022 were retrospectively analyzed. The patients were categorized into two groups based on the Glasgow Outcome Scale (GOS) 3 months after operation: poor outcome group (GOSI-III) and good outcome group (GOSIV and V). Multimodal monitoring included invasive intracranial pressure (ICP), brain temperature (BT), internal jugular venous oxygen saturation (SjvO_2_), and noninvasive NIRS. NIRS monitoring comprised the assessment of brain tissue oxygen saturation (StO_2_), blood volume index (BVI), and tissue hemoglobin index (THI). The prognostic differences between the two groups were compared. The predictive values were evaluated using the receiver operating characteristic (ROC) curve and the area under the curve (AUC).

**Results:**

ICP, BT, BVI, and THI in the good prognosis group were lower than those in the poor prognosis group. The SjvO_2_ and StO_2_ in the group with a good prognosis were higher than those in the group with a poor prognosis.

**Conclusion:**

The levels of ICP, BT, SjvO_2_, StO_2_, BVI, and THI reflect the changes in brain function and cerebral blood flow and significantly correlate with the prognosis of patients with SICH. NIRS monitoring has a high clinical utility in assessing the prognosis.

## 1 Introduction

Spontaneous intracerebral hemorrhage (SICH) refers to a hematoma resulting from the rupture of intracranial blood vessels due to nontraumatic factors (1). It is the second leading cause of stroke (2). The causes include primary SICH (hypertension and cerebral amyloid angiopathy) and secondary SICH (vascular malformations, nonatherosclerotic vascular lesions, tumors, coagulation disorders, and exposure to toxicants). The natural mortality rate within 1 month after the onset of SICH is approximately 45%, and approximately 80% of the patients have a poor prognosis (3, 4). Surgical treatment remains the primary treatment modality for SICH.

Although surgery has contributed to a reduction in the mortality rate among patients with SICH, the proportion of patients with poor prognosis in neurological function remains relatively high, which adds to the burden on patients’ families and the national health insurance system (5). There is still a lack of an effective treatment strategy to improve the prognosis of patients with SICH (6). Early and accurate detection of changes in intracranial conditions and timely and effective interventions can dramatically reduce the mortality of patients and substantially improve the prognosis. Near-infrared spectroscopy (NIRS) has been widely used in cardiac surgery to optimize the perfusion of the brain, heart, kidney, liver, and other important organs. However, its use in patients with SICH is limited. Clinicians can optimize oxygen delivery and possibly improve clinical outcomes by measuring brain tissue oxygen saturation (StO_2_) at the watershed of the anterior and middle cerebral arteries (7). By using multimodal monitoring that integrates intracranial pressure (ICP) with NIRS, this study enables real-time monitoring of intracranial conditions and offers precise treatment guidance for clinicians, thereby overcoming the limitations of empirical treatment approaches and providing an accurate assessment of the prognosis of patients.

## 2 Data and methods

### 2.1 Patient information and grouping

**Figure 1:**
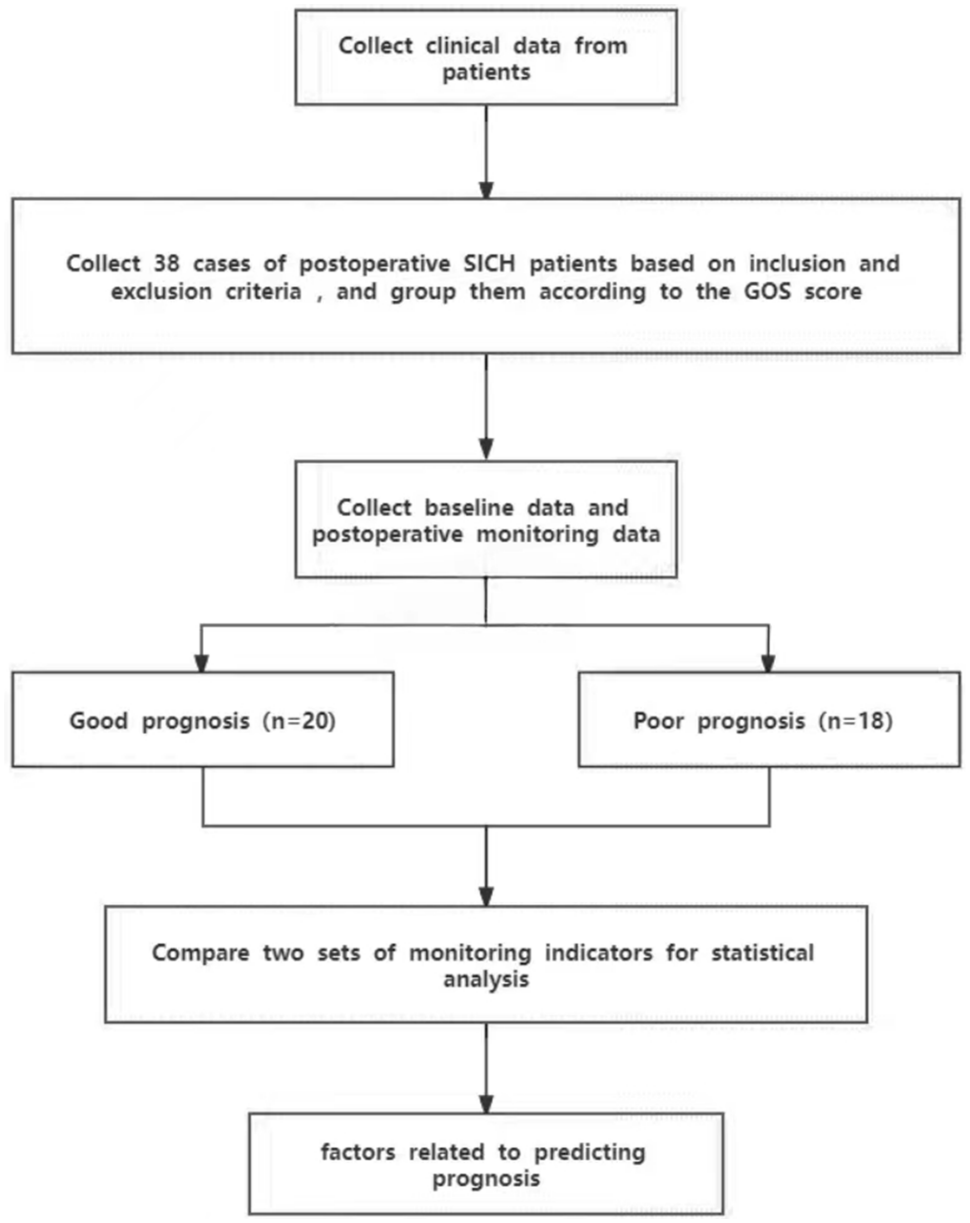
Flow chart for patients included in this study.

The clinical data of 38 patients with SICH who underwent surgery in the Department of Neurosurgery of Shaanxi Provincial People’s Hospital from May 2022 to December 2022 were retrospectively analyzed. The study sample included 17 male participants and 21 female participants (Figure 1). According to the guidelines (8), upon admission, all patients underwent emergency cranial CT examinations. This included dietary restrictions, oxygen inhalation, electrocardiogram (ECG) monitoring, assessment of consciousness, monitoring of basic vital signs, and optimization of relevant blood tests as part of preoperative preparation. The early systolic blood pressure of patients was maintained at <140 mm Hg to prevent the enlargement of hematoma and deterioration of the nervous system to promote functional recovery. The Glasgow Coma Scale (GCS) of the patients was between 4 and 9. The patients were categorized into two groups based on the Glasgow Outcome Scale (GOS) 3 months after discharge: poor outcome group (GOSI-III) and good outcome group (GOSIV-V). This study is in line with the principles of the Helsinki Declaration. An informed consent form for the surgery and placement of an ICP probe was signed by the families of all the patients.

The inclusion criteria for the study included the following: (1) relevant examination upon admission and presence of signs of cerebral hemorrhage based on the clinical symptoms and imaging findings; (2) indication of emergency surgery, including microscopic hematoma evacuation or neuroendoscopic minimally invasive surgery; (3) allowed to monitor ICP and brain temperature (BT); (4) allowed for the placement central venous catheter after surgery; (5) allowed assessing ICP, BT, and internal jugular venous oxygen saturation (SjvO_2_) and NIRS monitoring at least 3 days after the surgery.

The following patients were excluded from the study: (1) those with a history of head surgery; (2) those with multiple organ failure or other serious underlying diseases affecting the prognosis; (3) patients treated with anticoagulants or antiplatelet drugs for more than three months (due to the associated higher risk of poor prognosis); (4) those with a recurrent stroke within 3 months of follow-up after discharge; and (5) those who died of unknown causes within 24 h after surgery.

All patients underwent emergency surgery within 24 h after diagnosis, and all surgeries were performed by the chief physician or deputy chief physician at Shaanxi Provincial People’s Hospital. The surgical procedures included hematoma clearance and implantation of an ICP probe in the lateral ventricle. Bone flap decompression was performed in patients with severe brain swelling after hematoma clearance. The changes in dynamic ICP were monitored for continuously 3 days after the operation. All patients were transferred to an intensive care unit (ICU) and received standard treatment, including hemostasis, prevention of brain edema, nutritional support, and prevention of complications. Patients in a comatose state underwent tracheotomy to strengthen the management of the respiratory tract. The head CT was reexamined at 1, 3, and 7 days after the operation. ICP and BT were recorded every hour for the first three days post-operation. SjvO2 was monitored every eight hours for three days post-operation. NIRS monitoring was conducted every eight hours, for 20 min at a time, over the course of three days post-operation. The average values of ICP, BT, SjvO_2_, brain oxygen saturation (StO_2_), blood volume index (BVI), and tissue hemoglobin index (THI) were calculated.

Some specific treatments were administered as follows:

1. When the postoperative ICP was >22 mm Hg and lasted for >15 min, dehydration drugs (e.g., mannitol, furosemide, albumin) along with analgesic and sedative drugs were administered intravenously.
2. When the postoperative ICP was >30 mm Hg, intermittent and rapid administration of multiple dehydration drugs, cerebrospinal fluid drainage, analgesic and sedative therapy, and hyperventilation therapy were implemented.
3. When the postoperative ICP was >45 mm Hg, the head CT scan was reexamined and reoperation was considered based on the situation.
4. “Postoperative brain temperature management”: The BT was controlled at 37°C. However, in cases where the BT reached >37°C, an ice cap or ice blanket was used.

### 2.2 Data extraction

**Figure 2:**
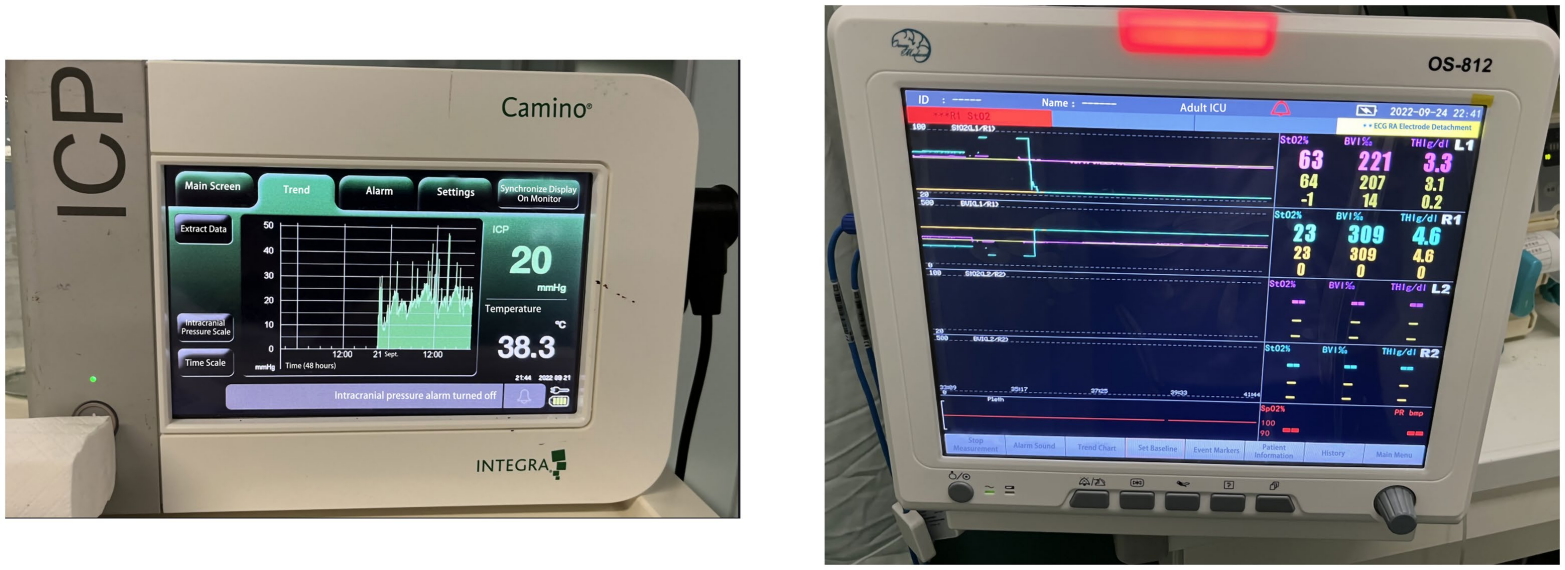
Monitoring equipment.

The ICP probe with BT monitoring was implanted into the ventricle of the patients after the surgery, and the probe was taken out after continuous monitoring for 3 days. The ICP and BT data stored on the monitor were collected, and the average values were calculated in an hour. A central venous catheter was placed in each patient. SjvO_2_ was measured every 8 h after the operation, and NIRS monitoring of 20 min was performed at the same time. The data of StO_2_, BVI, and THI stored on the monitor were collected and the average values were calculated (Figure 2).

### 2.3 Statistical analysis

SPSS software (version 25.0; IBM Corp) was used for statistical analysis. The Student *t*-test was used to compare the normally distributed data, and the data were expressed in terms of mean ± standard deviation. The Mann–Whitney U test was used to analyze non-normally distributed data between the groups, and the data were expressed using median (M) and interquartile range (IQR). Moreover, the classified variables were expressed by the number of cases and percentage or constituent ratio. The chi-square test and Fisher exact test were used for comparison. A *p*-value <0.05 was considered statistically significant. The MedCalc software (version 19.0.2) was used to prepare the subject receiver operating characteristic curve (ROC), and the area under the ROC curve (AUC) was calculated to evaluate the significance of each index in predicting the prognosis of patients.

## 3 Results

### 3.1 Comparison of baseline and cerebral oxygen metabolism indexes between the two groups

There was no significant difference in age, sex, and GCS score at admission between the good outcome group (*n* = 20) and the poor outcome group (*n* = 18). However, the average ICP and BT values in the good outcome group were significantly lower than those in the poor outcome group (ICP: 13.50 ± 5.03 vs. 25.11 ± 5.09; *p* < 0.001; BT: 36.96 ± 0.69 vs. 38.15 ± 0.78; *p* < 0.001). The SjvO_2_ (67.46 ± 4.89 vs. 53.11 ± 11.18, *p* < 0.001) and StO_2_ (66.39 ± 3.15 vs. 53.11 ± 17.08, *p* < 0.004) in the good outcome group were significantly higher than those in the poor outcome group. The values of BVI and THI in the poor outcome group were significantly higher than those in the good outcome group (BVI: 265.66 ± 94.27 vs. 199.05 ± 68.82, *p* = 0.019; THI: 4.03 ± 1.34 vs. 2.75 ± 0.61, *p* < 0.001) as shown in Table 1.

**Table 1:** Comparison of baseline and cerebral oxygen metabolism indexes between the two groups.

Influencing factors	Poor outcome group (18 cases)	Good outcome group (20 cases)	Result	*p*
Age (years) (≥60/<60)	7/11	11/9	χ^2^ = 0.986	0.253
Gender (Male/Female)	8/10	9/11	χ^2^ = 0.001	0.615
GCS score on admission M (IQR)	7 (4.75)	7 (6)	*Z* = 1.068	0.317
ICP (mmHg)	25.11 ± 5.09	13.50 ± 5.03	*t* = 7.059	<0.001
BT (°C)	38.15 ± 0.78	36.96 ± 0.69	*t* = 5.009	<0.001
SjvO2 (%)	53.11 ± 11.18	67.46 ± 4.89	*t* = 5.215	<0.001
StO2 (%)	53.11 ± 17.08	66.39 ± 3.15	*t* = 3.289	0.004
BVI (%)	265.66 ± 94.27	199.05 ± 68.82	*t* = 2.465	0.019
THI (g/dl)	4.03 ± 1.34	2.75 ± 0.61	*t* = 3.692	0.001

### 3.2 Comparison of different monitoring indexes for predicting poor prognosis of patients

**Figure 3:**
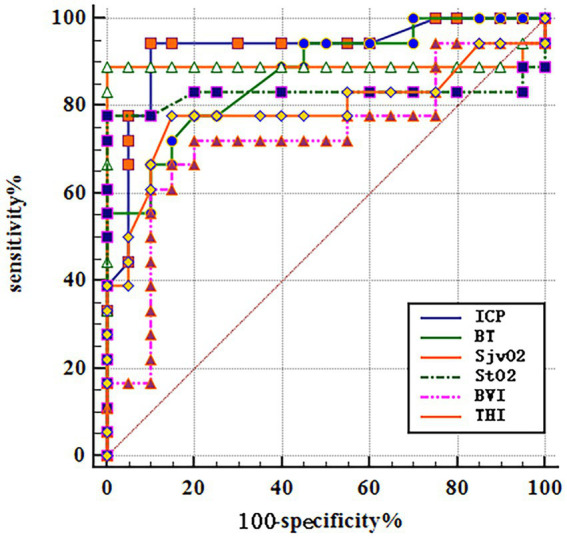
ROC curves for predicting the significance of different monitoring indicators in predicting poor prognosis in SICH patients based on relevant factors for predicting prognosis.

Analyze the significance of different monitoring indicators such as CP, BT, SjvO2, and NIRS for poor prognosis of patients with SICH after operation. The results showed that the average postoperative ICP (AUC = 0.928; 95%CI 0.795–0.986; *p* < 0.001), BT (AUC = 0.871; 95%CI 0.722–0.957; *p* < 0.001); SjvO_2_ (AUC = 0.892; 95%CI 0.748–0.969; *p* < 0.001); StO_2_ (AUC = 0.828; 95%CI 0.671–0.931; *p* < 0.001), BVI (AUC = 0.725; 95%CI 0.556–0.857; *p* < 0.001); and THI (AUC = 0.792; 95%CI 0.629–0.906; *p* < 0.001). Combined with multiple indexes, it is very important to predict the poor prognosis of patients with SICH as shown in Table 2 and Figure 3.

**Table 2:** The role of different monitor indexes in predicting the poor prognosis of patients with SICH.

Influencing factors	AUC	Sensitivity (%)	Specificity (%)	95%CI	*P*
ICP	0.928	94.44	90.00	0.795–0.986	<0.001
BT	0.871	77.78	80.00	0.722–0.957	<0.001
SjvO2	0.892	88.89	100	0.748–0.969	<0.001
StO2	0.828	77.78	100	0.671–0.931	<0.001
BVI	0.725	72.22	80.00	0.556–0.857	<0.001
THI	0.792	77.78	85.00	0.629–0.906	<0.001

## 4 Discussion

In recent years, a growing body of clinical evidence has demonstrated that multimodal neural monitoring, including parameters such as ICP, BT, and cerebral oxygen metabolism, plays a crucial role in mitigating the risks of secondary brain injury in individual patients and can serve as valuable predictors for assessing the prognosis after SICH (9, 10). Multimodal monitoring is an important reference index for the diagnosis and treatment of clinical neurosurgical diseases, offering valuable insights for the identification and determination of intracranial conditions. Multimodal monitoring can show the changes of various indexes continuously, dynamically, accurately, and intuitively, which has important guiding significance for the treatment of diseases with intracranial hypertension (11). The combined application of a variety of neural monitoring techniques to further integrate brain physiological data can precisely reflect the morbid response of the body, which is important for gaining a deeper understanding of the nature of the disease. Studies have shown that ICP, BT, and SjvO_2_ hold great importance in predicting the prognosis of TBI, and there are few studies assessing the use of NIRS in monitoring SICH. We aim to use this approach to predict the prognosis of patients after SICH, assessing its potential to improve prognosis and decrease mortality rates.

In recent years, the use of multimodal neural monitoring, including invasive methods such as ICP, BT, and SjvO2, has been suggested to be predictive of patient prognosis following SICH (12, 13). These methods, while highly accurate and specific, pose risks of complications like infection and thrombosis, and are not suitable for long-term monitoring (14). Our study explored the feasibility and advantages of NIRS as an alternative.

NIRS monitoring of StO2, BVI, and THI provides a noninvasive, continuous method to assess cerebral oxygenation and hemodynamics. In our study, NIRS-derived variables correlated well with patient outcomes, although their accuracy and specificity in predicting prognosis were slightly lower than those of invasive methods. Our findings suggest that NIRS can complement or potentially replace invasive methods in certain clinical scenarios. The absence of procedural complications and the capability for long-term monitoring are substantial advantages.

The NIRS technique is a powerful tool to evaluate brain autoregulation, vascular reactivity, and tissue oxygenation in patients. NIRS uses the transmission and absorption of near-infrared light in tissues to measure the relative ratio of oxyhemoglobin to deoxyhemoglobin. NIRS can distinguish complex hemodynamic patterns by simultaneously evaluating oxygenation and perfusion quality (StO_2_/BVI) parameters and oxygenated and deoxyhemoglobin (THI) distribution (15). StO_2_ measures cerebral oxygen content in mixed arteries and veins, which itself is affected by cerebral circulation, oxygen content, and oxygen extraction. Therefore, under the assumption that brain metabolism and oxygen transport are relatively constant, a fluctuation in StO_2_ reflects the local cerebral oxygen metabolism. Many studies have shown that a decrease of 20% or below to 60% in brain StO2 levels is associated with postoperative ischemic brain injury or re bleeding, and this threshold can serve as a reference indicator for clinical monitoring (16), which is consistent with the findings of our study. The average level of StO_2_ in the group with good prognosis was higher than that in the group with poor prognosis (66.39 ± 3.15 vs. 53.11 ± 17.08). In addition, among the three patients who died in the study, it was found that the significant difference in StO_2_ between bilateral cerebral hemispheres was associated with mortality. At present, there are no guidelines for measuring blood oxygen saturation in the brain tissues by NIRS. The guidelines issued by the Japanese Society of Cardiovascular Anesthesiologists highlight the efficacy of this noninvasive continuous monitoring technique in monitoring perfusion abnormalities during surgery. Our study showed that brain oxygen saturation monitoring was associated with the prognosis of patients, and the ROC curve showed an AUC of 0.828. It is of certain importance to predict the prognosis of patients with SICH. In the future, more research is required to determine the benefits of NIRS oxygenation monitoring in improving the outcomes related to the nervous system and other factors (17).

THI reflects tissue perfusion, and the increase in THI indicates cerebral vascular congestion (18). In our study, the level of THI in the poor prognosis group was higher than that in the good prognosis group (4.03 ± 1.34 vs. 2.75 ± 0.61). The increase in THI can be explained by the accumulation of red blood cells in the microcirculation of brain tissue, indicating perfusion disorders or rebleeding (19). The brain tissue is hypercongested, leading to reduced oxygen intake, thereby increasing the risk of recurrent cerebral hemorrhage and contributing to a poorer prognosis for the patient. The ROC curve showed an AUC of 0.792, indicating that THI in particular is of great importance in predicting the prognosis of patients. In this study, the BVI of the poor prognosis group was significantly higher than that of the good prognosis group (265.66 ± 94.27 vs. 199.05 ± 68.82; *p* = 0.019). High BVI in the poor prognosis group may be because of increased brain metabolism, increased cerebral blood flow, which may increase BT, and increased cerebral blood volume, which may increase ICP. Multiple factors interact with each other, resulting in poor neurological prognosis of patients. The ROC curve results showed that the AUC was 0.725, indicating that BVI had a potential utility in predicting the prognosis of SICH patients.

Our results suggest that NIRS monitoring techniques can be used to identify postoperative patients with SICH who exhibit persistent defects in brain tissue oxygenation and perfusion quality. The clinical relevance of StO_2_, BVI, and THI in different hemodynamic settings should be further evaluated in detail, as they may alert the brain tissue to underperfusion or overperfusion, causing irreversible neurological damage to the patient. Real-time noninvasive optical monitoring of tissue oxygenation levels through NIRS can provide meaningful clinical intervention to improve patient outcomes (20). NIRS could potentially meet ideal neurosurveillance requirements to detect brain tissue at risk of secondary damage and can complement or even replace the current invasive methods (21, 22). NIRS monitoring has several advantages including (1) noninvasive and real-time monitoring of abnormal brain tissue oxygenation status and (2) applicability in various scenarios, such as ICU, during transportation, or surgery (23, 24).

Multimodal monitoring can provide a therapeutic window for continuous prevention, early detection, and timely intervention of hypoxic/ischemic neuronal injury, thereby influencing clinical outcomes. Multimodal monitoring minimizes the limitations of each monitoring mode, and the components complement each other to improve the accuracy of the information obtained.

## 5 Limitations

This study is a single-center study, and the difference in surgical levels in different hospitals might have a certain impact on the prognosis of patients. Moreover, due to various reasons, we could only observe the monitoring indicators 3 days after surgery. In future studies, we should extend the observation time to improve the accuracy of prediction. Furthermore, this is a retrospective study with a small sample size, our study was not suitable for conducting multivariable regression analysis, making it unclear which of the six variables we researched are truly associated with a poor prognosis. Although our sample size is relatively small, it is also limited to single center studies. Due to the limited research on the use of NIRS for monitoring SICH, our research results demonstrate the feasibility of using NIRS for monitoring postoperative SICH patients, which has good research significance and can provide support for future multi center studies.

## 6 Conclusion

The goal of postoperative rehabilitation for neurocritical illness is to prevent or mitigate secondary brain injury through prediction, early detection, or monitoring of treatment response. The multimodal monitoring method, combining ICP with NIRS, uses real-time measurement of brain pathophysiology information to comprehensively understand and monitor the brain after injury. This method holds high predictive value, effectively guiding clinical decision-making and treatment methods; the timely intervention facilitated by this approach greatly improves the surgical prognosis of patients with SICH.

## Data availability statement

The original contributions presented in the study are included in the article/supplementary material, further inquiries can be directed to the corresponding author.

## Ethics statement

Ethical approval was not required for the study involving humans in accordance with the local legislation and institutional requirements. Written informed consent to participate in this study was not required from the participants or the participants’ legal guardians/next of kin in accordance with the national legislation and the institutional requirements.

## Author contributions

ZS: Writing – review & editing, Writing – original draft, Validation, Data curation. JL: Writing – review & editing. KW: Writing – review & editing. JZ: Writing – review & editing. SL: Writing – review & editing. FX: Writing – review & editing, Resources, Funding acquisition.
